# Association between acute kidney injury and mortality after successful cardiopulmonary resuscitation: a retrospective observational study

**DOI:** 10.1016/j.bjane.2021.02.026

**Published:** 2021-02-19

**Authors:** Ender Para, Mustafa Azizoğlu, Aslınur Sagün, Gülhan Orekici Temel, Handan Birbiçer

**Affiliations:** a Reyhanlı Devlet Hastanesi, Anesthesia and Reanimation Department, Hatay, Turkey; b Mersin University, Anesthesia and Reanimation Department, Mersin, Turkey; c Mersin University, Biostatistics and Bioinformatics Department, Mersin, Turkey

**Keywords:** Acute kidney injury, Post-cardiac arrest, RIFLE, KDIGO

## Abstract

**Background and objectives:**

Acute Kidney Injury (AKI) affect mortality and morbidity in critically ill patients. There have been few studies examining the prevalence of AKI and mortality after successful cardiopulmonary resuscitation. In the present study, we investigated the association between AKI and mortality in post-cardiac arrest patients admitted to the Intensive Care Unit (ICU).

**Methods:**

Our retrospective analysis included 109 patients, admitted to the ICU following successful cardiopulmonary resuscitation between 2014 and 2016. We compared two scoring systems to estimate mortality.

**Results and discussion:**

AKI were diagnosed in 46.7% (n = 51) of the patients based on the RIFLE criteria and 66.1% (n = 72) using the KDIGO. Mortality rate was significantly higher among patients with AKI diagnosed according to the RIFLE criteria (*p* = 0.012) and those with AKI diagnosed using KDIGO criteria (*p* = 0.003). Receiver Operating Characteristic (ROC) analysis showed that both scoring systems were able to successfully detect mortality (Area under the ROC curve = 0.693 for RIFLE and 0.731 for KDIGO).

**Conclusion:**

AKI increases mortality and morbidity rates after cardiac arrest. Although more renal injury and mortality were detected with KDIGO, the sensitivity and specificity of both scoring systems were similar in predicting mortality in patients with Return of Spontaneous Circulation (ROSC).

## Introduction

Acute Kidney Injury (AKI) is a clinical entity that can progress from a small rise in serum creatinine level to end-stage renal failure and is characterized by failure to eliminate urea and other nitrogenous waste from the body due to sudden loss of kidney function with resulting disruptions of extracellular fluid and electrolyte content.^1^ It usually occurs in patients in the Intensive Care Unit (ICU) due to toxic, ischemic, or obstructive causes and is associated with high mortality and morbidity. AKI has an incidence of 5–20% among patients admitted to the ICU, causing a 5-fold increase in morbidity and 35–65% mortality, regardless of patients’ comorbidities.^2^

The Risk, Injury, Failure, Loss, End-Stage (RIFLE), Acute Kidney Injury Network (AKIN), and Kidney Disease: Improving Global Outcomes (KDIGO) criteria are used in the diagnosis and classification of AKI.^3^ The RIFLE criteria classify AKI in five stages, while the KDIGO criteria classify AKI in three stages.^3^

After cardiac arrest, patients with Return of Spontaneous Circulation (ROSC) frequently have high mortality and morbidity rates due to cerebral, myocardial, and global ischemia-reperfusion damage. Post-cardiac arrest syndrome, which is a clinical presentation characterized by tissue hypoperfusion and multiple organ dysfunction, can lead to severe renal dysfunction.^4^ The incidence of AKI after cardiac arrest is 12–40%.^5^

Numerous studies have emphasized brain damage as the key factor determining life expectancy in patients with ROSC after successful cardiopulmonary resuscitation (CPR),^6^, ^7^ while the prevalence of extra-cerebral organ injury and its effects on mortality have received less attention.^8^

The aim of the present study was to analyze mortality rates in patients with ROSC who were diagnosed with acute kidney injury and/or failure based on RIFLE and KDIGO criteria. It can be estimated that, when using KDIGO, more patients can be diagnosed with AKI related to the criteria it contains. We hypothesized that KDIGO would be more helpful to demonstrate mortality than RIFLE. To the best of our knowledge, this is the first comparative study of RIFLE and KDIGO to predicting mortality in patients with ROSC after successful CPR in the literature.

## Methods

**Figure 1:**
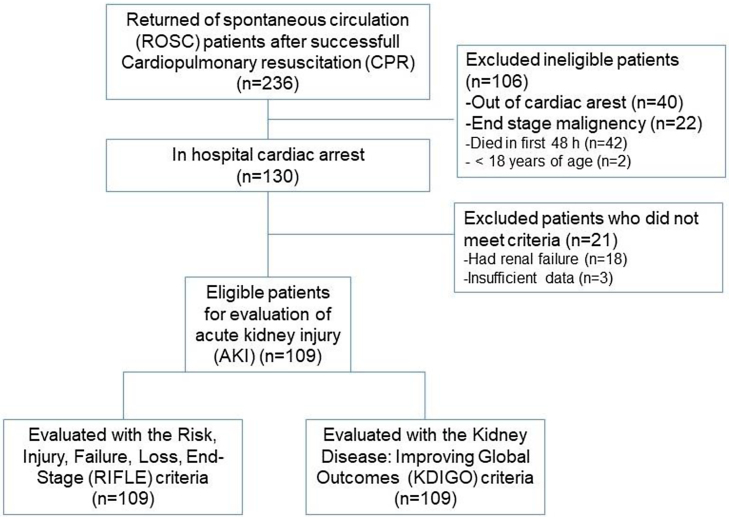
Flow diagram of the patient selection for comparison to acute kidney injury.

This retrospective observational study was performed in the ICU of the Mersin University Faculty of Medicine, Department of Intensive Care. After obtaining ethical approval from the Mersin University Clinical Studies Ethics Committee (14/04/2016, nº 2016/104), a total of 130 patients who had ROSC following in hospital cardiac arrest between 1 January 2014 and 28 February 2016 were retrospectively evaluated. Our intensive care unit is a “general intensive care unit” that receives patients requiring mechanical ventilation. All patients were already admitted to the hospital before cardiac arrest and were followed-up until discharge or death. All patients had cannulated radial or brachial artery during/after CPR. We retrospectively analyzed and documented the first measured Intra-Arterial Blood Pressure (IABP) after admission to the ICU. Patients with previously known renal pathology, end-stage malignancy, who were younger than 18 years, or died in the first 48 hours were excluded from the study. Patients with serum creatinine level greater than 1.5 mg.dL^-1^ were also excluded from the study. Data pertaining to the remaining 109 patients were obtained from ICU follow-up charts. A detailed flow diagram of the study is presented in Figure 1.

All patients were evaluated with both the RIFLE classification and KDIGO classification. In order to compare the diagnostic and staging capabilities of the two scoring systems, the RIFLE criteria for loss of kidney function (stage L) and end-stage renal failure (stage E) were included in the failure group (stage F) to yield a three-stage system consistent with the KDIGO classification.^3^ Data regarding the patients’ age, sex, cardiac arrest rhythm, Mean Arterial Pressure (MAP), resuscitation duration, admitting pH, serum creatinine levels, urinary output, and renal replacement therapy were obtained from their follow-up charts. Patients who survived 30 days after resuscitation were considered survivors. Survival/mortality data were obtained from daily follow-up forms for non-survivors or the electronic medical record system of the hospital for survivors. Creatinine levels and urinary output were checked individually according to the RIFLE and KDIGO criteria for each day beginning with the first admission. Our primary outcome was to compare renal failure detection rates the two systems and the 30-day mortality rates of patients with renal failure according to each classification. The secondary outcome was to compare survivors and non-survivors in terms of age, sex, duration of mechanical ventilation, initial cardiac ryhthm, duration of resuscitation, and admitting arterial blood pressure, creatinine level and serum pH.

The sample size was calculated using ClinCalc (https://clincalc.com/stats/samplesize.aspx). Based on data from our pilot study (of 15 renal injury patients evaluated, 50% those diagnosed with RIFLE and 70% of patients diagnosed with KDIGO died), a total of 93 patients were needed to reach 80% power with Type I error. SPSS 16.0 package software was used for statistical analysis. Normal distribution of the parameters in each group was assessed using Shapiro-Wilk test. For normally distributed parameters, mean and standard deviation were used as descriptive statistics; median and interquartile range (25^th^–75^th^percentile values) were given for parameters with non-normal distribution. Categorical variables were expressed as number and percentage values. Differences between two groups were evaluated using *t*-test for data with normal distribution and Mann-Whitney *U* test for those with non-normal distribution. Chi-Square test was used to evaluate the relationship between categorical variables. Receiver Operating Characteristic (ROC) analysis was also used to the predictive power of the two-scoring systems mortality. A *p*-value < 0.05 was considered statistically significant.

## Results

A total of 109 patients were included in the study (Figure 1). The mean age was 60.98 ± 20.1 years and there were 69 males and 40 females (63.3% vs. 36.7%). The demographic characteristics and clinical data of the patients are summarized in Table 1.

**Table 1:** Patient characteristics and clinical data of the patients.

Age (years)	60.98 ± 20.1
Sex (Male/Female)	69/40 (63.3% vs. 36.7%)
Mean duration of mechanical ventilation (days)	13.66 ± 15.90
Mean duration of resuscitation (min)	18.75 ± 11.71
Mean arterial pressure (mmHg)	77.66 ± 22.42
Number of discharged patients (n, %)	26 (23.9%)
Number of patients with shockable rhythm (ventricular fibrillation and pulseless ventricular tachycardia), n (%)	22 (20.1%)
Mean creatinine levels during admission (mg.dL^-1^)	1.32 ± 0.12
Number of patients treated with hemodialysis, n (%)	7 (6.3%)

The mean duration of resuscitation was 18.75 ± 11.71 minutes, and surviving patients had shorter resuscitation times (13.62 ± 13.58 min vs. 18.46 ± 13.42 min, *p* < 0.05). There was no difference in mean duration of resuscitation between patients diagnosed with kidney injury in both the KDIGO and RIFLE groups compared to patients who did not develop renal injury (17.74 ± 14.00 min vs. 17.40 ± 12.85 min with KDIGO, 18.43 ± 15.20 vs. 16.55 ± 12.01 for RIFLE; *p* > 0.05). MAP values at admission were significantly higher in surviving patients compared to non-survivors (86.38 ± 20.48 mmHg vs. 74.62 ± 22.48 mmHg, *p* < 0.05). No significant difference was detected between survivors and non-survivors in terms of duration of mechanical ventilation, or cardiac arrest rhythm (*p* > 0.05) (Table 2).

**Table 2:** Comparison to demographics and clinical data of the survivor and non-survivor patients.

	**Survivors**	**Non-survivors**	***p***
Age (year)	57.96 ± 19.92	60.98 ± 20.10	0.50
Sex (M/F)	14/12 (53.8%/46.2%)	55/28 (66.2%/33.8%)	0.22
Mean duration of mechanical ventilation (days)	7 (2–17.5)	6.5 (5–21)	0.67
Determined initial rhythm (shockable/non–shockable)	5/21	15/68	0.87
Mean duration of resuscitation (min)	13.62 ± 13.58	18.46 ± 13.42	0.02^a^
Mean Arterial Blood pressure (mmHg)	86.38 ± 20.48	74.62 ± 22.48	0.01^a^
Mean creatinine levels (mg.dL^-1^)	1.29 ± 1.05	1.34 ± 1.21	0.85
Mean blood pH	7.28 ± 0.13	7.20 ± 0.18	0.02^a^

a *p* < 0.05.

The mean age in the patient group diagnosed with AKI based on RIFLE criteria was 64.4 years, while the mean age in the patient group diagnosed with AKI based on KDIGO criteria was 63.8 years. In both systems, patients diagnosed as having renal injury had a significantly higher mean age than those without renal injury (KDIGO: 63.78 ± 18.56 vs. 52.89 ± 21.00 years, *p* < 0.05 and RIFLE: 64.47 ± 20.07 vs. 56.22 ± 19.30 years, *p* < 0.05).

When the presence and severity of renal injury were evaluated using the RIFLE and KDIGO criteria, a significantly greater number of patients were diagnosed with AKI based on KDIGO (n = 72, 66.1%) compared to RIFLE (n = 51, 46.7%) (Table 3) (*p* < 0.05).

**Table 3:** Comparison to development of acute kidney injury according to the RIFLE and KDIGO criteria.

	**Number of patients diagnosed with KDIGO (n = 72/109)**					***p***
**Number of patients diagnosed with RIFLE (n = 51/109)**		**Stage 0**	**Stage 1**	**Stage 2**	**Stage 3**	< 0.001^a^
	Stage 0	37 (33.9%)	21 (19.2%)	0	0	
	Stage 1	0	10 (9.1%)	1 (0.9%)	0	
	Stage 2	0	0	6 (5.5%)	6 (5.5%)	
	Stage 3–4–5	0	0	0	28 (25.6%)	

RIFLE, Risk, Injury, Failure, Loss, End-Stage, KDIGO, Kidney Disease: Improving Global Outcomes.

a *p* <  0.05.

**Figure 2:**
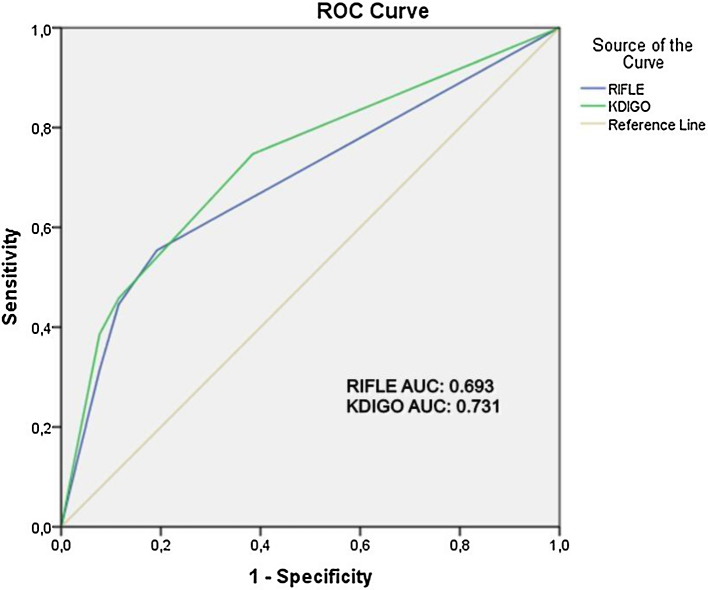
Receiver operating characteristics (ROC) Curve of predicting mortality of Risk, Injury, Failure, Loss, End-Stage (RIFLE) and Kidney Disease: Improving Global Outcomes (KDIGO). Area Under Curve (AUC) (RIFLE): 0.693, AUC (KDIGO): 0.731, *p* < 0.05. There was no difference between two system according to ROC curve analysis (*p* > 0.05).

The mortality rate was significantly higher among patients with AKI diagnosed according to the RIFLE criteria (*p* < 0.05) and those with AKI diagnosed using KDIGO criteria (*p* < 0.05) (Table 4). Mortality rates were similar in patients with stage 3 renal damage according to both scoring systems (32/34, 94% diagnosed with KDIGO vs. 26/28, 92% diagnosed with RIFLE; *p* > 0.05). In ROC analysis for mortality, Area-Under-Curve (AUC) was 0.693 for RIFLE and 0.731 for KDIGO (*p* < 0.05 for all). However, no statistically significant difference was found between RIFLE and KDIGO (*p* > 0.05) (Figure 2).

**Table 4:** Mortality rates of patients with acute kidney injury according to the RIFLE and KDIGO criteria.

	**Renal failure**	**Non-survivors**	**Survivors**	***p***
**Diagnosed with RIFLE criteria**	No	37 (33.9%)	21 (19.2%)	0.012^a^
	Yes	46 (42.2%)	5 (4.5%)	
	Stage 1	9 (8.2%)	2 (1.8%)	
	Stage 2	11 (10.0%)	1 (0.9%)	
	Stage 3–5	26 (23.8%)	2 (1.8%)	
**Diagnosed with KDIGO criteria**	No	21 (19.2%)	16 (14.6%)	0.003^a^
	Yes	62 (56.8%)	10 (9.1%)	
	Stage 1	24 (22.0%)	7 (6.3%)	
	Stage 2	6 (5.4%)	1 (0.9%)	
	Stage 3	32 (29.3%)	2 (1.8%)	

RIFLE, Risk, Injury, Failure, Loss, End-Stage; KDIGO, Kidney Disease: Improving Global Outcomes.

a *p* < 0.05.

## Discussion

This study showed that mortality rate after cardiac arrest was associated with the presence and severity of kidney injury evaluated using the RIFLE and KDIGO criteria. However, a larger proportion of patients were evaluated as having kidney injury when the KDIGO criteria were applied rather than RIFLE. AKI was diagnosed in 66.1% of the patients based on the KDIGO criteria, compared to 46.7% based on the RIFLE criteria. These results are consistent with the literature for both the RIFLE and KDIGO classifications.^9^ The fact that renal injury was more prevalent in our patients when using the KDIGO criteria seems to be related to its broader criteria for stage 1 compared to the RIFLE system.^10^ ROC analysis showed that both systems are effective in determining the risk of mortality.

In a study by Chua et al. evaluating 105 cardiac arrest patients, 33 (31.4%) were evaluated as having AKI or failure based on the RIFLE criteria.^11^ In a retrospective analysis of 82 cases of cardiac arrest cases by Kim et al., AKI was detected in 80.5% of the patients based on the KDIGO criteria.^12^ In the abovementioned studies, AKI staging was performed within the first 24–48 hours of admission to the ICU, and changes in AKI severity during ICU follow-up was not assessed. In our study, however, we took into account the highest creatinine level and/or the lowest urinary output of the patient within the first week of ICU follow-up when determining the AKI stage, which reflected the changes that occurred during follow-up.

In light of the literature and our results, it can also be stated that in addition to its better sensitivity in diagnosing AKI, the KDIGO classification is also a better estimation of mortality than RIFLE (mortality in patients diagnosed based on RIFLE vs. KDIGO: 55.4% vs. 74.7%).^10^

Whether the risk of developing AKI is associated with duration of resuscitation efforts has been debated in previous studies. Although several studies have shown that there was a significant difference in resuscitation duration between patients with and without AKI,^13^, ^14^ Chua et al. observed that resuscitation times were no longer in patients with AKI.^11^ In our study, comparison of patients with and without AKI revealed no significant difference in terms of duration of resuscitation. An experimental study conducted by Fu et al. also demonstrated that there is no relationship between resuscitation time and AKI.^15^

Tujjar et al. reported a higher mortality rate in patients who developed AKI (65%) compared to those who did not (50%).^13^ Consistent with the literature, there was a significant correlation in our study between mortality and kidney injury determined based on both the RIFLE and KDIGO criteria. Geri et al. analyzed the correlation between AKI development and mortality rate in 580 patients who had out-of-hospital cardiac arrest and found that stage 3 kidney injury was associated with significantly higher mortality rate.^14^ In our study, mortality rate increased progressively with degree of renal injury and we found 94% mortality rate in patients evaluated as stage 3 AKI based on the KDIGO criteria.

Yanta et al. stated that older age was associated with the development of AKI after cardiac arrest due to reduced physiological function of the kidneys.^16^ Similarly, we noted in our study that patients with AKI according to both the RIFLE and KDIGO criteria had higher mean ages than those without AKI.

Hasper et al. found that the mean creatinine level at initial post-cardiac arrest admission was 1.20 mg.dL^-1^.^17^ We determined a mean creatinine level of 1.32 mg.dL^-1^ in our study, and high serum creatinine level in particular was found to be significantly correlated with AKI diagnosis based on the KDIGO criteria.

In a review evaluating 1963 post-cardiac arrest patients, Sandroni et al. determined that non-shockable arrest rhythm, arrest duration, high serum creatinine level at admission, presence of post-resuscitative cardiogenic shock, and high serum lactate levels after resuscitation were factors in AKI development.^18^ A significant relationship between initial arrest rhythm or duration of resuscitation and AKI was not observed in our study. Hospital mortality was significantly higher in the group with AKI.

Burne Taney et al. stated that 10 minutes of ischemia-reperfusion was sufficient to cause elevation of serum creatinine levels, and a minimum duration of 30 minutes of renal artery clamping was required to increase serum creatinine levels to the same degree.^19^ In our study, the mean duration of resuscitation (time from lack of circulation to ROSC) was 17.7 minutes in patients diagnosed with AKI based on KDIGO criteria and 18.4 minutes in patients diagnosed with AKI based on RIFLE criteria, in concordance with experimental studies.

Numerous studies have focused on brain dysfunction as the main determinant of survival time in patients with ROSC after successful CPR, while there has been less research on the incidence of extra-cerebral organ injury and its impact on mortality. Our findings indicate that injury to various extra-cerebral organs, such as AKI, can also significantly increase the mortality rate.

This study has some limitations. Firstly, we conducted a retrospective study. We believe that a more reliable evaluation can be made with a prospective research. Secondly, we conducted a single-center study, and the sample size was too small for evaluations other than the primary outcome.

## Conclusion

In summary, AKI increases mortality rates after cardiac arrest. More renal injury and mortality can be detected with KDIGO, but the success of the two methods is similar in predicting mortality in patients after resuscitation. More studies are needed to determine the best method to predict mortality in patients after successful CPR.

## Conflicts of interest

The authors declare no conflicts of interest.
